# Fatty Acid Transfer from Blood to Milk Is Disrupted in Mothers with Low Milk Production, Obesity, and Inflammation

**DOI:** 10.1093/jn/nxac220

**Published:** 2022-10-08

**Authors:** Rachel E Walker, Kevin J Harvatine, A Catharine Ross, Erin A Wagner, Sarah W Riddle, Alison D Gernand, Laurie A Nommsen-Rivers

**Affiliations:** Department of Nutritional Sciences, The Pennsylvania State University, University Park, PA, USA; Department of Animal Science, The Pennsylvania State University, University Park, PA, USA; Department of Nutritional Sciences, The Pennsylvania State University, University Park, PA, USA; College of Allied Health Sciences, University of Cincinnati, Cincinnati, OH, USA; Division of Hospital Medicine, Cincinnati Children's Hospital Medical Center, Cincinnati, OH, USA; Department of Pediatrics, University of Cincinnati School of Medicine, Cincinnati, OH, USA; Department of Nutritional Sciences, The Pennsylvania State University, University Park, PA, USA; College of Allied Health Sciences, University of Cincinnati, Cincinnati, OH, USA

**Keywords:** low milk supply, lipoprotein lipase, fatty acids, inflammation, lactation, TNF-α

## Abstract

**Background:**

Obesity is associated with chronic inflammation and is a risk factor for insufficient milk production. Inflammation-mediated suppression of LPL could inhibit mammary uptake of long-chain fatty acids (LCFAs; >16 carbons).

**Objectives:**

In an ancillary case–control analysis, we investigated whether women with low milk production despite regular breast emptying have elevated inflammation and disrupted transfer of LCFAs from plasma into milk.

**Methods:**

Data and specimens from a low milk supply study and an exclusively breastfeeding control group were analyzed, with milk production measured by 24-h test-weighing at 2–10 wk postpartum. Low milk supply groups were defined as very low (VL; <300 mL/d; *n* = 23) or moderate (MOD; ≥300 mL/d; *n* = 20) milk production, and compared with controls (≥699 mL/d; *n* = 18). Serum and milk fatty acids (weight% of total) were measured by GC, serum and milk TNF-α by ELISA, and serum high-sensitivity C-reactive protein (hsCRP) by clinical analyzer. Group differences were assessed by linear regression models, chi-square exact tests, and Kruskal–Wallis nonparametric tests.

**Results:**

VL cases, as compared with MOD cases and controls, had higher prevalence of elevated serum hsCRP (>5 mg/L; 57%, 15%, and 22%, respectively; *P* = 0.004), detectable milk TNF-α (67%, 32%, and 33%, respectively; *P* = 0.04), and obesity (78%, 40%, and 22%, respectively; *P* = 0.003). VL cases had lower mean ± SD LCFAs in milk (60% ± 3%) than MOD cases (65% ± 4%) and controls (66% ± 5%) (*P* < 0.001). Milk and serum LCFAs were strongly correlated in controls (*r* = 0.82, *P* < 0.001), but not in the MOD (*r* = 0.25, *P* = 0.30) or VL (*r* = 0.20, *P* = 0.41) groups (*P*_int_ < 0.001).

**Conclusions:**

Mothers with very low milk production have significantly higher obesity and inflammatory biomarkers, lower LCFAs in milk, and disrupted association between plasma and milk LCFAs. These data support the hypothesis that inflammation disrupts normal mammary gland fatty acid uptake. Further research should address impacts of inflammation and obesity on mammary fatty acid uptake for milk production.

## Introduction

Breastfeeding confers multiple health benefits to mothers and infants, and both the American Academy of Pediatrics and Healthy People 2030 have targeted increased breastfeeding as a public health goal (1). Healthy People 2030 reports that about three-quarters of women do not meet the recommendation of exclusive breastfeeding for the first 6 mo (2). Although nearly 80% of women in the United States initiate breastfeeding, the number of mothers meeting breastfeeding recommendations at 6 mo drops to 25% (2, 3). Among mothers who stop breastfeeding earlier than intended, low milk production is one of the most common reasons cited (4, 5).

In the United States, ∼40% of reproductive age women (20–39 y) have obesity (6). Lactating mothers with obesity are at increased risk of poor lactation outcomes (7–10). Insulin resistance and other markers of poorer metabolic health are associated with delayed lactogenesis and low milk production (11–16). It is often difficult to disentangle physiologic mechanisms impairing lactation from the effects of behavioral differences in breastfeeding that may covary with obesity or associated conditions. Physiologic mechanisms and potential treatments of low milk production in humans are understudied (4, 17).

Obesity is a cause of chronic low-grade inflammation, which leads to a marked elevation of acute-phase proteins and inflammatory cytokines, such as TNF-α (18). Elevated plasma TGs are a marker of insulin resistance and the metabolic syndrome, resulting partly from the suppression of LPL expression in adipose tissue by inflammatory cytokines such as TNF-α (18–20). However, it is unknown how inflammation and obesity may alter mammary lipid metabolism in lactating mothers.

**FIGURE 1:**
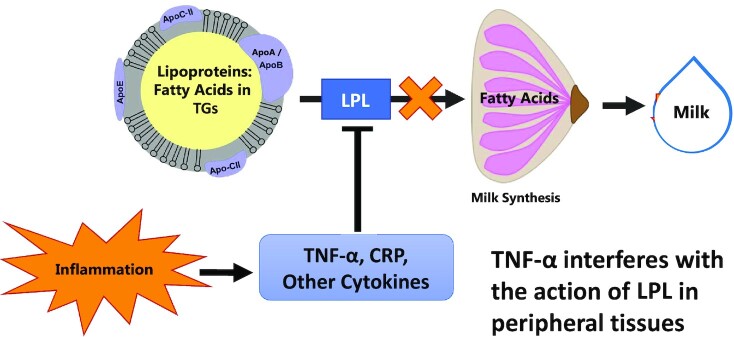
Hypothetical model for reduced milk production as a result of inflammation-mediated suppression of mammary LPL. Our working model used to build the hypotheses in this project was that inflammatory cytokines such as TNF-α suppress mammary LPL, leading to less fatty acid available to the mammary gland. This would be reflected in lower concentrations of LCFAs (>16 carbons) in milk, because nearly all LCFAs in milk are derived from the circulation by LPL. In addition, less LCFA is available to the mammary gland for energy production, reducing the milk synthesis rate. LCFA, long-chain fatty acid.

Our previous work found that plasma TGs were higher in mothers with physiologically very low milk production than in mothers with moderately low to normal and adequate production (16). LPL activity in the mammary epithelial cell basal membrane is a regulated and controlled step in the transport of fatty acids from maternal circulation into the mammary gland (21, 22). The mammary gland requires fatty acids as an energy source and as a substrate for synthesis of TGs in the milk fat globule. Because TNF-α inhibits LPL expression in other tissues (19), chronic inflammation as evidenced by elevated C-reactive protein (CRP) and TNF-α secretion may be a metabolic mechanism leading to disrupted mammary fatty acid transfer from plasma and contributing to insufficient milk production (**Figure 1**).

In humans, dietary fat intake strongly predicts milk fatty acid profiles (23, 24), and there is a strong relation between circulating and milk concentrations of many fatty acids, especially the long-chain PUFAs (25–28). If LPL in the mammary gland is suppressed by chronic inflammation, the normally strong relation between circulating and milk PUFAs would be altered. This could explain altered milk fatty acid profiles reported in mothers with overweight and obesity compared with mothers who are lean (29, 30). Nearly all long-chain fatty acids (LCFAs; >16 carbons) are derived from the circulation, whereas 16-carbon fatty acids are derived from mixed sources, and a large portion of the mid-chain fatty acids (MCFAs; <16 carbons) in milk are produced by mammary de novo lipogenesis (25, 31). Therefore, higher proportional LCFAs in milk indicate higher transfer of fatty acids from plasma to milk, and lower proportional MCFAs indicate lower incorporation of fatty acids from mammary de novo lipogenesis.

Based on our clinical findings and the known effects of chronic inflammation on systemic lipid metabolism, we hypothesize that serum and milk fatty acid profiles will differentiate physiologically low milk production from healthy milk production. Thus, in this study, we aimed to test this hypothesis by analyzing biorepository serum and milk fatty acid profiles and inflammatory markers obtained from 61 lactating women, including 23 with very low milk production despite frequent breast emptying. First, as an indicator of de novo and preformed fatty acid uptake in the mammary gland, we compared the relative concentrations of MCFAs (<16 carbons) and LCFAs (>16 carbons) according to maternal milk production status, and second, we examined if the relation between serum and milk fatty acid profiles is modified by maternal milk production status and markers of inflammation. Because of the mixed sources of diet-derived and endogenously synthesized 16-carbon fatty acids in milk, we did not develop a specific hypothesis related to their concentrations even though they were measured.

## Methods

### Study design and subjects

**FIGURE 2:**
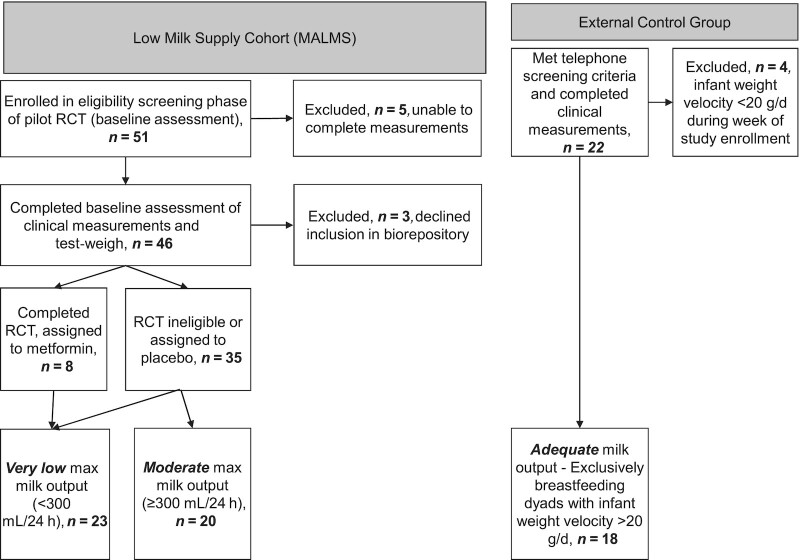
Flowchart for inclusion in the ancillary study. Participants were recruited to a study of low milk supply (*n* = 46), but 3 declined participation in future analysis. A small number (*n* = 8) were assigned to metformin treatment as part of a pilot clinical trial. All participants in the low milk supply study were categorized by milk production as very low (<300 mL/d; *n* = 23) or moderate (≥300 mL/d; *n* = 20). External controls were recruited who were exclusively breastfeeding infants with healthy weight gain (*n* = 18). RCT, randomized controlled trial.

We conducted an observational, ancillary case–control study using data and specimens previously collected as part of a low milk supply study (32) and from an external control group (33) recruited at Cincinnati Children's Hospital Medical Center and the University of Cincinnati, respectively (**Figure 2**). Enrollment into the original studies is briefly described here. First, all participants in the low milk supply study were referred by area lactation support services or self-referred for low milk supply because they were supplementing with infant formula despite a desire to exclusively breastfeed. These mothers were assessed at baseline for inclusion into a 4-wk pilot randomized controlled trial using metformin to augment low milk supply (clinicaltrials.gov: NCT02179788; NCT02179788I) (32). In addition, participants who completed baseline measurements for the metformin trial but who decided to not enroll into the trial or who did not meet strict selection criteria were invited to participate in an observation-only follow-up arm. Inclusion criteria for the low milk supply study baseline assessment were 1–8 wk postpartum, ≥20 y of age, and healthy singleton infant born at ≥37 weeks of gestation. Participants were excluded if they were feeding or pumping <6 times per breast in a 24-h period, unwilling to continue frequent breast emptying for 2–4 wk, lived outside the defined geographical area, lacked established pediatric care for the infant, had a history of breast surgery, had a diagnosis of type 1 or type 2 diabetes, had a current nipple or breast infection, or were currently using metformin. An International Board Certified Lactation Consultant (IBCLC) visited all participants for a comprehensive home visit, including counseling to optimize frequent and thorough breast emptying. Milk production was measured at baseline, 2 wk, and 4 wk later.

All participants in both arms of the low milk supply study were considered for inclusion in this ancillary analysis. We grouped the low milk supply study participants into very low milk production (VL) and moderate milk production (MOD) groups based on their maximal milk output from test-weigh measurements over the course of follow-up. We defined cases of VL based on milk output never exceeding 300 mL/d during any test-weigh measurement period (range: 0–295 mL/d), which is <50% of the minimum intake of an exclusively breastfed infant (16, 34). We categorized participants in the low milk supply study with milk production ≥300 mL/d during ≥1 test-weigh measurement period as MOD (range: 305–835 mL/d). Although all participants in the MOD group self-reported low milk supply and were supplementing with formula at baseline, a wide range of milk production volumes were observed over the course of their study participation. Maximal milk production was >700 mL/d (range: 738–835 mL/d) for 4 participants and >600 mL/d (range: 634–835 mL/d) for 6 participants in the MOD group. Eight low milk supply study participants were assigned to metformin treatment, and all 8 were in the VL group with milk production <300 mL at baseline and throughout the trial. Their mean ± SD baseline milk output was 139 ± 75 mL/d, and their mean increase in milk production was 21 ± 61 mL/d between baseline and final test-weigh, with 6 of these participants having their maximum milk output at the final point (i.e., after metformin treatment).

Low milk supply study participants provided a milk sample at baseline and 2 and 4 wk postbaseline during a research clinic visit after a fasting blood sample was taken. Approximately 1–3 h after the last breastfeeding event, a 10-mL milk sample was collected at the end of a full breast emptying session, with the intention of sampling hindmilk. If milk production was very low (∼10 mL/feed on each breast according to the most recent test-weigh), the entire pumped milk was used for this sample. For this analysis, we used the milk and serum sample (baseline or 4 wk) that corresponded with time of maximal milk production for that subject. If the sample from maximal production was unavailable, an alternative time point was used (2 wk; *n* = 3), because the milk production group classification did not change for any of the participants.

Second, we included data and specimens from an external control group of mothers who were successfully exclusively breastfeeding with adequate production, based on their infant exhibiting a healthy rate of weight gain (>20 g/d; referred to as “controls” throughout the article). Controls were enrolled under a separate protocol based on demographic inclusion criteria similar to those of the low milk supply study (33): 4–10 wk postpartum at time of screening (to align with the low milk volume study's 2- to 4-wk follow-up time frame), maternal age ≥ 20 y, and currently exclusively breastfeeding a healthy singleton infant born at ≥37 weeks of gestation. External controls were excluded if they lived outside the defined geographic area, had a history of breast surgery, had a diagnosis of type 1 or type 2 diabetes, had a history of insufficient infant weight gain, or were tandem breastfeeding (breastfeeding an older sibling simultaneously). Participants were further excluded if infant weight gain was <20 g/d during an initial enrollment period. Thus, all participants from the external control group had adequate milk production to support healthy weight gain in their exclusively breastfed infant. External control subjects provided a milk sample at a research clinic visit after fully emptying breasts by pumping exactly 1 h after a baseline breastfeeding/breast emptying session. Fasting serum samples were also obtained for external control subjects at the same research clinic visit.

For both the low milk supply study and controls, anthropometric measurements were taken at Cincinnati Children's Nutrition Core to determine BMI (in kg/m^2^). Staff measured height (±0.1 cm) and weight (±0.2 kg) in duplicate, and repeated measures in the case of a >0.1-cm discrepancy for height or a >0.2-kg discrepancy for weight (16). Serum and milk samples were stored in a biorepository at −80°C for 2–5 y before the current analysis. Further excluded from this analysis were participants who refused inclusion of their data and specimens into a biorepository for secondary analyses at the time of consent or who were missing milk or serum samples for another reason.

### Ethical considerations

The low milk supply study protocols were approved by the Institutional Review Board (IRB) at Cincinnati Children's Hospital (IRB#: 2012-2333) (33), and the study protocols for the external controls were approved by the University of Cincinnati IRB (IRB#: 2016-8465) (32). All study participants gave written informed consent before initiation of any study procedures and had the option to agree to inclusion of their samples in a biorepository for use in future studies. All samples and data were deidentified before transfer to The Pennsylvania State University for the current study. Protocols for the current study were reviewed by The Pennsylvania State University IRB and designated as non–human research because new participants were not recruited and participants in the previous trial could not be identified (IRB#: 00015304).

### Milk production measurement

Milk production was measured by at-home test-weighing, as reported previously (16). Each participant was trained in the test-weigh protocol during a home visit (33, 35). Participants were provided with an infant scale (±2 g; Tanita BD-815U) to measure milk production by recording of infant or milk container weight before and after feeding or pumping. Participants in the low milk supply study continued test-weighing for 24 h. As part of a milk volume methodology and validation protocol, the external controls with adequate production continued test-weighing for 48 h. Test-weigh measurements were checked closely for plausibility and accuracy and test-weigh measurements that were suspect were repeated on a different day. Milk production was measured in 12 of the 18 exclusively breastfeeding external controls included in this analysis (range: 699–1334 mL/d). The remaining 6 exclusively breastfeeding external controls were screened for adequate infant growth, but did not complete the test-weigh protocol.

### Serum inflammatory biomarker analysis

TNF-α was measured in duplicate in serum using a commercially available ultra-sensitive ELISA assay with a limit of detection of 0.5 pg/mL (Invitrogen, Thermo Fisher; product # KHC3014). Samples were analyzed in duplicate according to the manufacturer's instructions. The TNF-α ELISA had a mean ± SD intra-assay CV within duplicates of 4% ± 4% and interassay CV of 4% for quality control samples.

Serum high-sensitivity CRP (hsCRP) was measured by the Biomarker Core Laboratory at The Pennsylvania State University on a Cobas c 311 clinical analyzer (Roche Diagnostics; product # 07876033 190). This analysis uses an immunoturbidimetric assay kit with standard clinical quality control protocols checked before each assay, with a limit of detection of 3 mg/L. Serum samples were available for hsCRP analysis for 21 out of 23 participants in the VL production group.

### Milk TNF-α analysis

Milk TNF-α concentrations were measured in duplicate using an ultra-sensitive ELISA assay with a limit of detection of 0.5 pg/mL (KHC3014; Invitrogen, Thermo Fisher). Because this assay was designed and validated for use in human serum and plasma, we verified the reliability of the assay in human milk using a TNF-α spike in 2 milk samples. In addition, we compared the assay results in whole milk with those in aqueous (de-fatted) milk from the same milk sample. We observed consistently lower absorption in the whole milk samples than in the aqueous samples, so aqueous samples were exclusively used for this analysis (**Supplemental Figure 1**). Although many previous studies did not report which milk fraction was used for TNF-α analysis by ELISA, several reported using the aqueous fractions (36–38). Aqueous milk samples were analyzed in duplicate according to the manufacturer's instructions with a mean ± SD intra-assay CV within duplicates of 9% ± 9%. TNF-α was undetected in the quality control milk sample used for this analysis, so interassay reliability could not be assessed, except to confirm that undetectable status was consistent across batches. Milk samples were unavailable for TNF-α analysis in 3 participants, reducing sample sizes to the following: VL, *n* = 21; MOD, *n* = 19; and controls, *n* = 18.

### Serum and milk fat profile analysis

Milk samples were unavailable for fat analysis for 3 participants in the VL production group and 1 participant in the MOD group. Therefore, sample sizes for milk fat analyses were as follows: VL, *n* = 20; MOD, *n* = 19; and controls, *n* = 18. Milk total fat concentration was measured gravimetrically by modified Folch extraction (39, 40). First, 0.5 mL of whole milk was extracted twice in 3 mL chloroform, using 1.5 mL methanol to precipitate proteins, and 2 mL 0.7% sodium chloride to separate the organic phase. Excess water was absorbed from the organic phase using 1–2 g anhydrous sodium sulfate, and extracts were decanted through a 10-μm PTFE mesh filter (Mitex, Millipore Sigma) into preweighed aluminum weighing pans. Extracts were left to dry overnight and weighed to the nearest milligram the next morning to determine total milk fat (g/mL). Measurements were reliable with an interassay CV of 5% in control samples between batches.

Milk fatty acid profile was obtained by GC with flame ionization detection (GC-FID; Agilent 6890A, Agilent Technologies) after a base-hydrolyzed methylation of esterified milk fatty acids as previously described (41). Briefly, 200 μL of whole milk was extracted in 2.5 mL of 3:2 (vol:vol) hexane:isopropanol mixture and 1.2 mL of 7% sodium sulfate solution. Fatty acids in hexane were methylated using 10 μL 1 M sodium methoxide in methanol at room temperature for 8 min with 10 μL methyl acetate added to minimize artifacts from the saponification reaction. The reaction was terminated using 100 μL of a termination reagent (oxalic acid, 30 mg/mL, in diethyl ether). Excess methanol and water were removed from the sample using calcium chloride before analysis. FAMEs dissolved in hexane were then transferred to autosampler vials and 2 μL of each sample was measured by GC-FID.

Serum fatty acid profiles were analyzed by 2-step hydrolysis and methylation of fatty acids followed by detection by GC-FID (41, 42). Lipids were extracted from 100 μL of the serum sample with 50 μg each of tridecanoic acid (13:0) and methyl nonadecanoic acid (19:0) as internal standards. In 8-mL glass extraction tubes, 1 mL of 3:2 (vol:vol) hexane:isopropanol and 0.5 mL of pH 3.0 citrate phosphate buffer were added to samples and mixed on a vortex for 1 min. Tubes were centrifuged at 1200 × *g* for 5 min at 4°C and lipids dissolved in the top hexane layer were transferred to a new tube with ∼0.25 g sodium sulfate crystals and allowed to sit for 30 min to finalize precipitation of serum proteins. The hexane layer was transferred to a new tube, dried under nitrogen, and reconstituted in 0.5 mL toluene. Serum fatty acids were first hydrolyzed and methylated by transesterification with 1 mL 0.5 M sodium methoxide in methanol for 10 min at 50°C. Because sodium methoxide is a poor methylating reagent for NEFAs, samples were methylated again with 1.5 mL 5% methanolic hydrochloric acid for 10 min at 80°C. The methylation reaction mixture was neutralized with 3.75 mL 6% potassium carbonate in water. Finally, tubes were centrifuged at room temperature at 300 ×   *g* for 5 min and FAMEs were extracted in 1 mL heptane. Samples were then dried and FAMEs were reconstituted in 300 μL heptane. Extracted samples (2 μL) were injected onto the GC-FID and analyzed by GC-FID using a fused-silica capillary column [SP-2560; 100 m × 0.25 mm (i.d.) with 0.2-μm film thickness; Supelco].

Peaks were identified using pure methyl ester standards (GLC 566 and 780; NuChek Prep Inc.) and measured using OpenLab software (2017, Agilent Technologies). Response factors were calculated to determine recoveries of individual fatty acids and calculate concentrations as weight percentage of total fatty acids using an equal-weight reference standard (GLC 461; NuChek Prep Inc.). Fatty acids that were consistently observed at ≥0.05% were included in the calculation. Fatty acids were categorized based on chain length as follows: MCFAs (<16 carbons), 16-carbon fatty acids, and LCFAs (>16 carbons); and quantified as weight percentage of total fatty acids by summing the weight percentages for all individual fatty acids in each chain length category. In serum, there were 28 total fatty acids detected, with 3 fatty acids included in the MCFA category, 3 in the 16-carbon category, and 22 in the LCFA category. The mean ± SD intra-assay CVs between serum duplicates for MCFAs, 16-carbon, and LCFAs were 5% ± 5%, 0.8% ± 1%, and 0.3% ± 0.3%, respectively. In milk, there were 40 total fatty acids detected, with 8 fatty acids included in the MCFA category, 3 in the 16-carbon category, and 29 in the LCFA category. The mean ± SD intra-assay CVs between milk duplicates for MCFAs, 16-carbon, and LCFAs were 2% ± 3%, 0.7% ± 2%, and 0.5% ± 0.5%, respectively.

### Statistical methods

All variables were examined for distribution, missingness, and outliers. Demographic and birth characteristics measured as continuous variables (maternal age, infant gestational age, and day postpartum) were all normally distributed and thus compared across the 3 milk production groups using 1-factor ANOVA. Dichotomized demographic and birth characteristics (ethnicity, college graduate, parity, gestational diabetes, vaginal delivery, infant sex, time point of milk sample) were compared by milk production group using Pearson chi-square test or Fisher's exact test in instances of cell sizes ≤ 5.

Group differences in BMI and serum TNF-α were compared across milk production groups using Kruskal–Wallis rank sum tests. BMI was also dichotomized as obesity (>30) and nonobesity (<30). Milk TNF-α was dichotomized as undetected (samples below the limit of detection: 0.5 pg/mL) or detected. Serum hsCRP was dichotomized as <5 mg/L and >5 mg/L, a common cutoff used in studies of inflammation (43, 44). Differences in dichotomized variables were compared by milk production group using the Pearson chi-square test or Fisher's exact test in instances of cell sizes ≤ 5.

Differences in individual fatty acid concentrations (weight% of total fatty acids) were compared across the 3 milk production groups using 1-factor ANOVA with post hoc Bonferroni pairwise comparisons. For each of the 3 fatty acid chain length categories (MCFAs, 16-carbon, and LCFAs), linear models were constructed to predict milk fatty acid weight percentage from serum fatty acid weight percentage, with milk production group and its interaction term (milk production group × serum fatty acid weight percentage) included in each model, using the controls as the reference group.

In order to rule out any effect of the underlying pilot randomized controlled trial, a sensitivity analysis was performed. Each outcome model was repeated without metformin-treated subjects and again with only metformin-treated subjects in the VL group. Similarly, each outcome model was repeated with only the control subjects with test-weigh milk production measurements (*n* = 12). Statistical analyses were performed using Stata version 17.0 (StataCorp, LLC) and JMP Pro version 15.0 (SAS Institute Inc.).

## Results

All mothers delivered full-term infants; the majority were primiparous and delivered vaginally. Mothers in the VL group (<300 mL/d) were not statistically different in age, demographics, and birth outcomes to those in the MOD group (≥300 mL/d) and controls (≥699 mL/d). Breast emptying frequency differed between milk production groups, with the MOD group emptying their breasts significantly more often than controls (**Table 1**).

**TABLE 1:** Sociodemographic, pregnancy, and delivery characteristics of mothers with very low milk production, moderate milk production, and controls^1^

	Milk production group^2^			
	VL (*n* = 23)	MOD (*n* = 20)	Control (*n* = 18)	*P* value^3^
24-H milk production, mL/d	162 ± 87^c^	520 ± 172^b^	822 ± 172^a,3^	<0.001
Maternal age, y	31 ± 6.3	32 ± 3.4	31 ± 4.7	0.58
Ethnicity, white	91	95	N/A	1.00
College graduate	65	85	89	0.18
Parity, primiparous	57	45	33	0.33
Gestational diabetes	22	5	6	0.21
Vaginal delivery	52	80	78	0.09
Gestational age at birth, wk	39 ± 0.9	39 ± 1.2	40 ± 1.1	0.11
Infant sex, male	43	45	39	0.93
Days postpartum at baseline	31 ± 18	33 ± 16	N/A	0.72
Days postpartum at milk sample	41 ± 18	43 ± 23	52 ± 12	0.14
Baseline time point of milk sample^4^	65	70	N/A	0.74
Breast emptying frequency,^5^ times/d	18 ± 6^a,b^	20 ± 8^a^	14 ± 4^b^	0.048

1 Values are mean ± SD or percentages. Group differences for normally distributed data were analyzed by 1-factor ANOVA with Bonferroni adjustment for repeated measures. Group means in a row with different superscript letters were significantly different (*P* < 0.05). Dichotomous variable differences were assessed by Pearson chi-square test. MOD, moderate milk production group; N/A, not available; VL, very low milk production group.

2 Milk production groups were defined as VL (<300 mL/d), MOD (≥300 mL/d), and control (externally recruited controls who were exclusively breastfeeding infants with healthy weight gain).

3 For the control group, *n* = 12 for milk production measured by test-weigh.

4 We used samples from the time point with maximal milk output from the low milk supply study (baseline or 4 wk). Controls were only assessed at 1 time point.

5 Breast emptying frequency was based on each breast counted separately.

The VL milk production group differed in BMI and inflammatory biomarkers, compared with the MOD and control groups. Mean BMI was >10 units higher for the VL than for the MOD and control groups (*P* < 0.001), and more than three-quarters of VL mothers had BMI > 30 (**Table 2**). In addition, mothers in the VL group were 2 times more likely to have detectable TNF-α in milk (*P* = 0.04) and more likely to have hsCRP > 5 mg/L (*P* = 0.004) (Table 2). There was no significant difference in serum TNF-α between groups.

**TABLE 2:** BMI and inflammatory markers of mothers with very low milk production, moderate milk production, and controls^1^

	Milk production groups^2^			
	VL (*n* = 23)	MOD (*n* = 20)	Control (*n* = 18)	*P* value
BMI,^3^ kg/m^2^	39.6 [31.3–41.8]^a^	29.0 [25.3–31.2]^b^	26.1 [21.6–28.2]^b^	<0.001
Obesity, % BMI ≥ 30 kg/m^2^	78	40	22	0.003
Inflammatory markers				
Serum hsCRP,^4^ % >5 mg/L	57	15	17	0.004
Serum TNF-α,^5^ pg/mL	6.3 [5.8–7.0]	6.1 [5.7–6.7]	5.8 [5.3–6.5]	0.31
Milk TNF-α,^6^ % detected	67	32	33	0.04
Milk TNF-α concentration of detected samples,^7^ pg/mL	1.60 [0.88–2.13]	0.98 [0.77–2.49]	1.11 [0.70–1.42]	N/A

1 Values are median [IQR] or percentages. Group differences were analyzed by nonparametric Kruskal–Wallis rank sum tests. Group median values in a row with different letters were significantly different (*P* < 0.05). Proportional differences were assessed by Pearson chi-square test. CRP, C-reactive protein; hsCRP, high-sensitivity CRP; MOD, moderate milk production group; VL, very low milk production group.

2 Milk production groups were defined as VL (<300 mL/d), MOD (≥300 mL/d), and control (externally recruited controls who were exclusively breastfeeding infants with healthy weight gain).

3 BMI measured at the baseline time point for VL and MOD groups.

4 Serum samples were unavailable for 2 participants: VL, *n* = 21; MOD, *n* = 20; control, *n* = 18.

5 One outlier of serum TNF-α > 20 pg/mL was removed for this analysis: VL, *n* = 23; MOD, *n* = 19; control, *n* = 18.

6 Milk samples were unavailable for TNF-α analysis for 3 participants: VL, *n* = 21; MOD, *n* = 19; control, *n* = 18. The lower limit of detection was 0.5 pg/mL.

7 Milk TNF-α concentrations are provided for the samples with a detectable value (>0.5 pg/mL): VL, *n* = 14; MOD, *n* = 6; control, *n* = 6.

**FIGURE 3:**
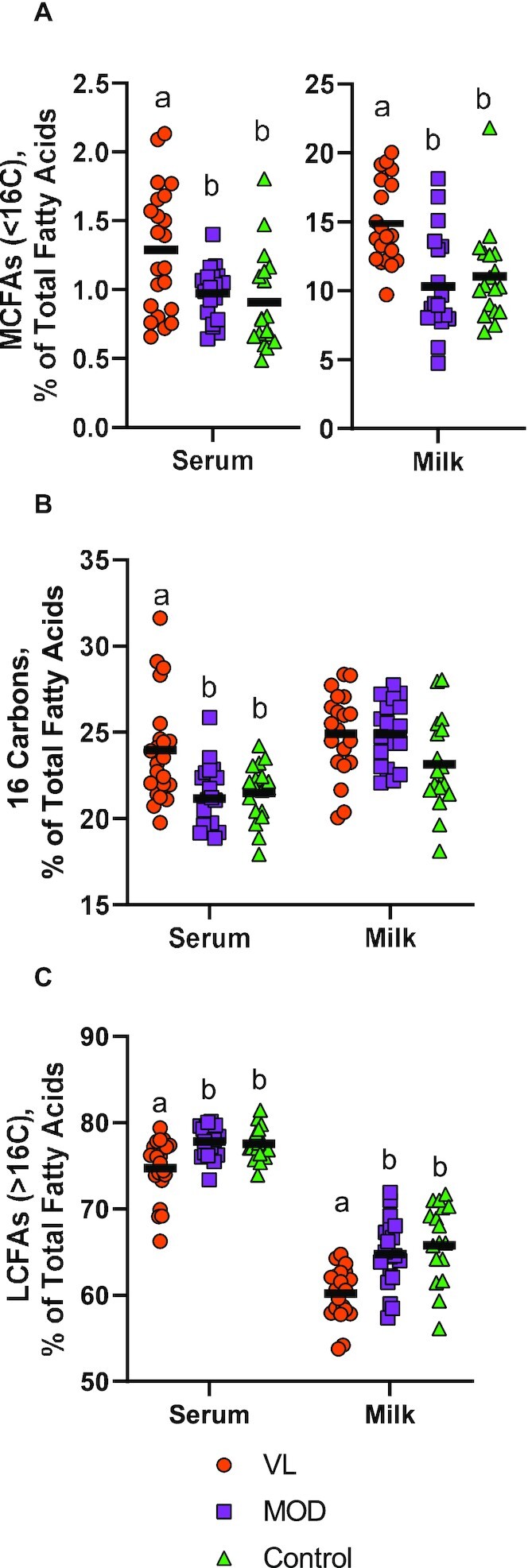
Differences in fatty acid profile of mothers with very low milk production, moderate milk production, and controls. Milk production groups were defined as VL (<300 mL/d), MOD (≥300 mL/d), and control (externally recruited controls who were exclusively breastfeeding infants with healthy weight gain). Individual values (shapes) and group means (horizontal lines) are shown for both serum and milk for (A) MCFAs (<16 carbons), (B) 16-carbon fatty acids, and (C) LCFAs (>16 carbons). Differences in means were assessed using 1-factor ANOVA with Bonferroni adjustment for multiple comparisons. Group means with different letters were statistically significantly different (*P* < 0.05). A serum sample was unavailable for 1 participant: VL, *n* = 22; MOD, *n* = 20; and control, *n* = 18. Milk samples were unavailable for fat analysis for 4 participants: VL, *n* = 20; MOD, *n* = 19; and control, *n* = 18. LCFA, long-chain fatty acid; MCFA, mid-chain fatty acid; MOD, moderate milk production group; VL, very low milk production group.

Serum and milk fatty acid profiles differed significantly by milk production groups. Mothers in the VL group had higher mean ± SD MCFAs (<16 carbons) in both serum (1.3% ± 0.5%) and milk (15% ± 3%) than MOD mothers (serum: 0.97% ± 0.2%, *P* = 0.02; milk: 10% ± 4%, *P* < 0.001) and controls (serum: 0.91% ± 0.4%, *P* = 0.004; milk: 11% ± 3%, *P* = 0.003). In contrast, the VL group exhibited significantly lower mean ± SD LCFAs (>16 carbons) in both serum (75% ± 3%) and milk (60% ± 3%) than mothers in the MOD group (serum: 78% ± 2%, *P* = 0.001; milk: 65% ± 4%, *P* = 0.002) and controls (serum: 78% ± 2%, *P* = 0.003; milk: 66% ± 5%, *P* < 0.001) (**Figure 3**). **Supplemental Tables 1** and **2** and **Supplemental Figure 2** report all differences in serum and milk fatty acid profiles. Mean ± SD milk total fat was significantly higher in controls (5.5 ± 1.8 g/dL) than in VL mothers (3.6 ± 1.9 g/dL; *P* = 0.01) (Supplemental Table 2). However, these differences should be interpreted with caution, because milk sampling protocols differed between the low milk supply study and controls.

**FIGURE 4:**
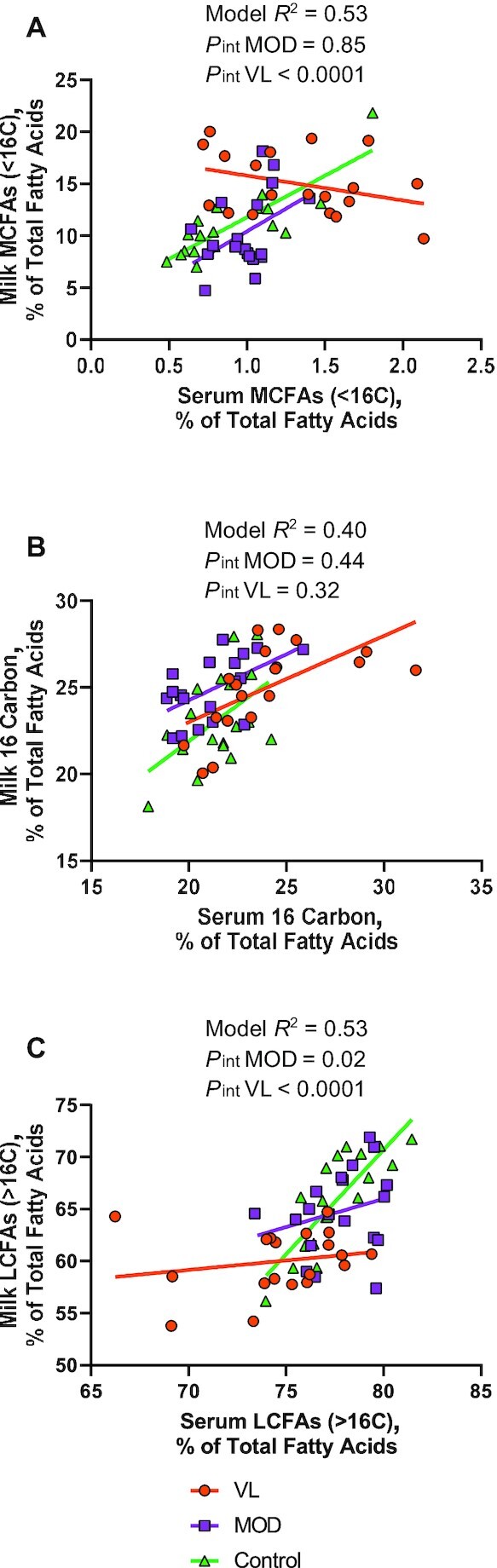
Association between serum and milk fatty acid profiles of mothers with very low milk production, moderate milk production, and controls. Milk production groups were defined as VL (<300 mL/d), MOD (≥300 mL/d), and control (externally recruited controls who were exclusively breastfeeding infants with healthy weight gain). Serum fatty acids (exposure) were used to predict milk fatty acids (outcome) using linear regression. Production group, and serum fatty acid-by-group interaction, were included in the model to assess differences in the association by milk production. In the interaction analysis, the VL and MOD milk production groups were compared with the controls as the reference group. Model results are shown for (A) MCFAs (<16 carbons), (B) 16-carbon fatty acids, and (C) LCFAs (>16 carbons). A serum sample was unavailable for 1 participant and milk samples were unavailable for fat analysis for 4 participants: VL, *n* = 19; MOD, *n* = 19; and control, *n* = 18. LCFA, long-chain fatty acid; MCFA, mid-chain fatty acid; MOD, moderate milk production group; VL, very low milk production group.

An interaction in the relation between serum and milk fatty acid profiles was observed by milk production group for both MCFAs (<16 carbons) and LCFAs (>16 carbons) (**Figure 4**). Only controls had a significant positive correlation between serum and milk fatty acids for MCFAs (*r* = 0.84, *P* < 0.001) and LCFAs (*r* = 0.82, *P* < 0.001). Correlations between serum and milk MCFAs and LCFAs were not statistically significant for either the MOD (MCFAs, *r* = 0.44, *P* = 0.06; LCFAs, *r* = 0.25, *P* = 0.30) or the VL (MCFAs, *r* = −0.34, *P* = 0.15; LCFAs, *r* = 0.20, *P* = 0.41) production group. Results of these models were not changed with sensitivity analysis including and excluding metformin-treated participants, ruling out confounding from the original pilot randomized controlled trial. Similarly, results remained unchanged when excluding control participants with no test-weigh milk production measurement. Based on these sensitivity analyses, our significant results appeared to be robust in this sample.

## Discussion

In this study, we found that the serum and milk fatty acid profiles and inflammatory markers differed in the VL group despite regular breast emptying compared with the MOD group or exclusively breastfeeding controls. Specifically, the VL group had higher BMI and inflammatory markers along with lower relative concentrations of milk LCFAs (>16 carbons), consistent with the hypothesized mechanism that inflammation suppresses the transfer of fatty acids from circulation to milk. In addition, we observed a strong association between serum and milk fatty acid profiles in control mothers, but not in the VL and MOD groups. These analyses provide strong evidence that insufficient transfer of fatty acids from circulation to the mammary gland is contributing to physiologically low milk production in women with obesity and inflammation.

The connection between obesity and poor breastfeeding outcomes is well established (7–10). Our finding of increased obesity prevalence in the VL group supports previous work that has reported lower milk transfer to the infant (45) and higher rates of perceived insufficient milk supply (46) in women with obesity than in those with normal BMI. Obesity is related to an increased risk of delayed secretory activation (7) and shorter duration of any and exclusive breastfeeding in various cohorts (9, 10, 47). Although this is generally attributed to social and mechanical barriers to breastfeeding for mothers with obesity, potential physiologic mechanisms linking obesity to poor lactation outcomes are understudied. Limited evidence indicates that prenatal metabolic health predicts delayed onset of mammary secretory activation (13) and mothers with obesity have a lower prolactin response (48). However, physiologic predictors of low milk volume have not been identified. In this study, we showed that mammary lipid metabolism is altered in mothers with low milk production. Specifically, control mothers who were all exclusively breastfeeding showed a tight association between LCFAs in circulating serum and LCFAs in milk, but this association disappeared in the VL group. This suggests a physiologic barrier to the transfer of fatty acids from blood to the mammary gland.

This study was not designed to establish physiologic mechanisms of low milk supply, but our results are consistent with some known mechanisms of disrupted lipid metabolism. TNF-α, specifically, was discovered in the context of its potent suppressive effect on LPL activity (18, 19). TNF-α-mediated suppression of LPL has been clearly demonstrated in the adipose tissue (20, 49, 50), and is associated with clinically elevated TGs in patients with diabetes (51). In addition to elevated inflammation and obesity, mothers with very low milk production in this study had significantly elevated plasma TGs (16). Elevated plasma TGs are associated with insulin resistance and chronic inflammation (52), and may be caused by decreased plasma clearance from suppressed LPL activity (53–56). LPL activity is critical to the function of the mammary gland (31). During lactation, the mammary gland is a primary energy sink in the body, using ∼20% of all energy intake (57). Therefore, even a moderate suppression of mammary LPL will result in large fluctuations of energy usage and fatty acid clearance in a lactating woman. Suppression of mammary LPL by chronic systemic inflammation, or inflammation localized to the breast, should be investigated further as a mediating factor in the etiology of low milk production (58). Animal models have demonstrated that TNF-α and the TNF-α receptor play an active role in regulation of the development of the mammary gland (59, 60). Therefore, it is possible that chronic inflammation, especially localized to the breast, is working during both pregnancy and lactation to alter milk production through mammary development and energy availability.

To date, very few studies have investigated the connection between inflammation and milk production in humans. However, subclinical mastitis is a globally prevalent condition of localized breast inflammation that is associated with both elevated milk concentrations of inflammatory cytokines (61) and impaired infant growth (62, 63). Our work may provide an explanatory mechanism for this impaired growth due to insufficient fatty acid uptake by the mammary gland. Another recent study showed very high rates of perceived insufficient milk supply in women with inflammatory bowel disease, but it is unclear if these rates are different than expected in the population (64).

Previous studies have shown changes in fatty acid profile with obesity. Several studies have reported higher ω-6 PUFA and decreased ω-3 PUFA in milk from mothers with obesity compared with normal BMI (30, 65), and 1 study observed lower concentrations of select milk MCFAs with obesity (29). Maternal BMI may also affect concentrations of certain specialized fatty acid metabolites in milk (66). To our knowledge, differences in milk fatty acid profile with low milk production have not been reported.

This study has shown clear physiologic alterations in lipid metabolism in mothers with very low milk production. This underscores the urgent need for more patient-centered holistic research in this area in order to elucidate the biological mechanisms leading to low milk production (67). Currently, the majority of mothers who stop breastfeeding before they intended cite low milk production as one of the primary reasons (5). However, tests for or treatments of low milk volume are lacking with the exception of frequent and thorough breast emptying (68). Failure of the mammary gland to produce sufficient milk represents a medical condition leading to negative health outcomes for both mothers and children. These health effects are important in high resource settings and high-income countries, but they are profound with devastating consequences in low resource settings and low- and middle-income countries with unpredictable access to clean water and high-quality infant formula (69, 70). Therefore, low milk production is a medical condition that has disproportional effects in vulnerable populations, making it an important contributor to global and domestic health disparities. Future studies are needed to clarify the true prevalence of low milk production in diverse populations in the United States and around the world.

There is growing recognition that low breastfeeding rates are associated with adverse long-term maternal health, including increased risk of breast cancer and cardiovascular disease (71, 72). However, in this work, short breastfeeding duration is generally assumed to be the causal factor. It is possible that poor metabolic health and chronic inflammation during pregnancy lead to poor mammary development and suppression of mammary LPL, making low milk production a potential early warning sign for future disease. Future studies should consider physiologically low milk production as a key opportunity for assessing disease risk.

### Strengths and limitations

One of the most important strengths of this study was the confirmation of milk production by measurement of 24-h milk volume in the low milk supply study and 48-h milk volume measurements in the external controls. Many studies have reported breastfeeding outcomes, but very few have objectively measured milk volume and breast emptying frequency. Therefore, we were able to verify true cases of very low milk production (VL; <300 mL/d) compared with moderate milk production (MOD; ≥300 mL/d) and exclusively breastfeeding external controls with healthy infant weight gain. Another important strength was the intensive coaching and lactation support provided to all participants in the low milk supply study by an IBCLC.

This study also had some important limitations, including a small sample size. This was an ancillary analysis with a case–control design in a small group of participants. The sample recruited for this study was not very diverse, with the majority of participants being white and college graduates. These results should be replicated in larger, prospectively enrolled cohorts in diverse settings in order to improve generalizability. Longitudinal cohorts beginning during pregnancy would also improve our understanding of risk factors that predict low milk production. The collection method of milk samples also limits our interpretation of milk fat results. For example, external controls provided their sample from a full pumping session 1 h after a previous full pumping session. In contrast, mothers in the low milk supply study provided a hindmilk sample at the end of a pumping session. This means that total milk fat results should be interpreted with caution, because hindmilk samples can have a fat content as much as 3-fold higher than foremilk (25). In this study, we limited our analysis to milk fatty acid profiles reported as percentage of total fatty acids, which do not differ between fore- and hindmilk (73). Stage of lactation and time postpartum can also affect total milk fat and milk fatty acid profile, but these changes primarily reflect differences between colostrum and transitional/mature milk (74–79). Although we had a range of timing (days postpartum), there were no significant differences between the groups in days postpartum of the milk sample. The generalizability of our results was also limited by confounding in our case–control study, because both obesity and inflammatory biomarkers were strongly associated with cases of VL. Therefore, it is not possible to determine if inflammation is associated with disrupted mammary fatty acid transfer independently from obesity in this sample.

### Conclusions

In this study, we observed meaningful physiologic differences in lipid metabolism in mothers with verified very low compared with moderate or adequate milk production, despite exclusive breastfeeding intention and frequent breast emptying. Specifically, we found that the proportional concentration of LCFAs (>16 carbons) was lower and the proportional concentration of MCFAs (<16 carbons) was higher in milk from the VL group. In addition, the strong association between serum and milk LCFAs observed in the control group disappeared in the VL group. Our data suggest that there is a disruption in the transfer of circulating fatty acids to the mammary gland for milk synthesis, likely due to suppression of mammary LPL activity by chronic inflammation. Overall, our data support the hypothesis that inflammation in lactating mothers leads to disrupted mammary transfer of fatty acids from circulation to the mammary gland and low milk volume. Future studies should be designed to examine fatty acid transfer to milk and the relation with milk volume.

## Supplementary Material

nxac220_Supplemental_File

## Data Availability

Data described in the article, code book, and analytic code will be made available upon request pending approval of a data sharing agreement.

## Notes

Supported by NIH grants NIH 5 K12 HD051953 (to LAN-R) and NIH 5UL1TR001425-03 (to LAN-R); USDA National Institute of Food and Agriculture grant 2020-67034-31767 (to REW); and the Dorothy Foehr Huck Endowment (to ACR).

Author disclosures: The authors report no conflicts of interest.

Supplemental Figures 1 and 2 and Supplemental Tables 1 and 2 are available from the “Supplementary data” link in the online posting of the article and from the same link in the online table of contents at https://academic.oup.com/jn/.

ADG and LAN-R contributed equally to this work as co–senior authors.

Abbreviations used: CRP, C-reactive protein; FID, flame ionization detection; hsCRP, high-sensitivity CRP; IBCLC, International Board Certified Lactation Consultant; IRB, Institutional Review Board; LCFA, long-chain fatty acid; MCFA, mid-chain fatty acid; MOD, moderate milk production group; VL, very low milk production group.
