# Supplementary material for: Fatty Acid Transfer from Blood to Milk Is Disrupted in Mothers with Low Milk Production, Obesity, and Inflammation
Source: J Nutr. 2022 Oct 8;152(12):2716–26. doi: 10.1093/jn/nxac220 (PMC9840005; doi:10.1093/jn/nxac220)
Supplement: nxac220_Supplemental_File [file nxac220_supplemental_file.docx]

**Supplemental Material**

Because the Invitrogen ELISA kit for TNF-α (KHC3014; Invitrogen, Thermo Fisher; Carlsbad, CA) had been validated for human serum but not human milk, we analyzed the reliability of replication and recovery of this ELISA using our milk samples. We chose one sample high in TNF-α concentration and one with detectable, but low TNF-α concentration. We performed at least two replicates of aqueous (de-fatted) and whole milk aliquots for each sample and found a mean intra-assay CV of approximately 4% for both aqueous and whole milk. Whole milk aliquots were lower in concentration than aqueous aliquots for both the low and high TNF-α samples. However, this difference was more dramatic (28% reduction) in the high TNF-α sample compared with the low TNF-α sample (6% reduction; Supplemental Figure 1A).

Aqueous samples containing either high or low TNF-α were then spiked with 12 pg/mL of TNF-α and subjected to the ELISA assay. Both spiked samples resulted in at least 80% recovery of the TNF-α spike. The high TNF-α sample had a higher recovery (96%) compared to the low TNF-α sample (82%; Supplemental Figure 1B).

**Supplemental Figure 1. Results of validation experiment investigating reliability of human serum TNF-α ELISA assay in human milk.** A) TNF-α concentrations were lower in whole milk compared to aqueous, especially in the high TNF-α sample. B) TNF-α spike recovery was >80% for both aqueous samples, but was higher in the high TNF-α sample. TNF-α, tumor necrosis factor α. Aq, aqueous milk aliquot. Wh, whole milk aliquot.

**Supplemental Table 1. Serum Fatty Acid Profiles**

|  | Milk Production Groups^1^ | | |  |
| --- | --- | --- | --- | --- |
| % of Total Fatty Acids  Mean (SD) | VL | MOD | Control | p-value |
| C12:0 | 0.11 (0.06)^a^ | 0.07 (0.03)^b^ | 0.07 (0.04)^b^ | 0.008 |
| C14:0 | 0.98 (0.40)^a^ | 0.71 (0.15)^b^ | 0.67 (0.29)^b^ | 0.004 |
| C15:0 | 0.20 (0.05) | 0.19 (0.04) | 0.17 (0.05) | 0.10 |
| C16:0 | 20.1 (2.5)^a^ | 18.1 (1.2)^a^ | 18.2 (1.3)^a^ | 0.001 |
| C16:1t n-7 | 0.42 (0.09)^a^ | 0.38 (0.07)^a^ | 0.29 (0.04)^b^ | <0.001 |
| C16:1 n-7 | 3.46 (0.91)^a^ | 2.63 (0.87)^b^ | 2.99 (0.64)^a,b^ | 0.007 |
| C17:0 | 0.24 (0.06) | 0.24 (0.04) | 0.21 (0.02) | 0.08 |
| C18:0 | 6.54 (0.94) | 6.86 (0.59) | 6.90 (0.66) | 0.25 |
| C18:1t | 0.89 (0.31)^a^ | 0.80 (0.40)^a^ | 0.55 (0.14)^b^ | 0.003 |
| C18:1 n-9 | 19.6 (2.8)^a^ | 17.5 (2.06)^b^ | 17.5 (1.76)^b^ | 0.004 |
| C18:1 n-7 | 1.51 (0.31) | 1.42 (0.21) | 1.37 (0.15) | 0.16 |
| C18:1 n-6 | 0.14 (0.06)^a^ | 0.14 (0.06)^a^ | 0.07 (0.03)^b^ | <0.001 |
| C18:2 n-6 | 33.0 (4.1)^b^ | 36.9 (3.7)^a^ | 38.9 (3.6)^a^ | <0.001 |
| C18:3 n-6 | 0.10 (0.04) | 0.08 (0.03) | 0.09 (0.04) | 0.39 |
| C18:3 n-3 | 0.13 (0.04)^a^ | 0.12 (0.03)^a,b^ | 0.10 (0.02)^b^ | 0.01 |
| C20:0 | 0.62 (0.26) | 0.52 (0.21) | 0.49 (0.13) | 0.13 |
| C20:1 n-9 | 0.69 (0.17)^a^ | 0.63 (0.13)^a,b^ | 0.56 (0.10)^b^ | 0.01 |
| C20:2 n-6 | 0.19 (0.07) | 0.20 (0.06) | 0.16 (0.04) | 0.09 |
| C20:3 n-6 | 1.50 (0.36)^a^ | 1.45 (0.34)^a^ | 1.15 (0.18)^b^ | 0.002 |
| C20:4 n-6 | 7.09 (1.89) | 8.08 (1.58) | 7.36 (1.07) | 0.12 |
| C20:5 n-3 | 0.46 (0.16) | 0.54 (0.20) | 0.41 (0.16) | 0.08 |
| C22:4 n-6 | 0.19 (0.09) | 0.19 (0.08) | 0.17 (0.04) | 0.82 |
| C22:5 n-6 | 0.18 (0.12) | 0.18 (0.07) | 0.15 (0.05) | 0.62 |
| C22:5 n-3 | 0.34 (0.07)^a,b^ | 0.37 (0.08)^a^ | 0.31 (0.06)^b^ | 0.04 |
| C22:6 n-3 | 1.13 (0.45)^a,b^ | 1.44 (0.51)^a^ | 1.05 (0.37)^b^ | 0.02 |
| C24:0 | 0.07 (0.05) | 0.08 (0.05) | 0.06 (0.03) | 0.61 |
| C24:1 n-9 | 0.10 (0.09)^a^ | 0.10 (0.07)^a^ | 0.04 (0.03)^b^ | 0.008 |

^1^Milk production groups were defined as very low (VL): <300 mL/d, moderate (MOD): > 300mL/d, and Control: exclusively breastfeeding infants with healthy weight gain.

^2^Group differences for were analyzed by one-factor ANOVA with *post hoc* Bonferroni pairwise comparisons. Group median values with different letters were statistically different (p<0.05).

**Supplemental Table 2. Milk fatty acid profiles**

|  | Milk Production Groups^1^ | | |  |
| --- | --- | --- | --- | --- |
| % of Total Fatty Acids  Mean (SD) | VL | MOD | Control | p-value |
| C6:0 | 0.05 (0.03)^b^ | 0.04 (0.03)^b^ | 0.07 (0.02)^a^ | 0.002 |
| C8:0 | 0.16 (0.07)^a,b^ | 0.13 (0.07)^b^ | 0.20 (0.04)^a^ | 0.006 |
| C10:0 | 1.42 (0.24)^a^ | 1.14 (0.42)^b^ | 1.30 (0.30)^a,b^ | 0.03 |
| C12:0 | 6.06 (1.43)^a^ | 3.85 (1.71)^b^ | 4.27 (1.46)^b^ | <0.001 |
| C14:0 | 6.68 (1.57)^a^ | 4.60 (1.48)^b^ | 4.67 (1.71)^b^ | <0.001 |
| C14:1 n-5 | 0.20 (0.06) | 0.22 (0.07) | 0.20 (0.09) | 0.68 |
| C15:0 | 0.29 (0.07) | 0.32 (0.07) | 0.31 (0.12) | 0.46 |
| C16:0 | 22.1 (2.17) | 21.8 (1.87) | 20.6 (2.56) | 0.10 |
| C16:1t n-7 | 0.49 (0.09)^a,b^ | 0.55 (0.08)^a^ | 0.45 (0.06)^b^ | 0.003 |
| C16:1 n-7 | 2.35 (0.56)^a,b^ | 2.59 (0.54)^a^ | 2.08 (0.47)^b^ | 0.02 |
| C17:0 | 0.30 (0.05) | 0.33 (0.06) | 0.31 (0.06) | 0.19 |
| C17:1 n-8 | 0.21 (0.04) | 0.23 (0.05) | 0.21 (0.05) | 0.14 |
| C18:0 | 6.65 (1.20) | 6.66 (1.09) | 6.82 (1.09) | 0.86 |
| C18:1t n-10-12 | 0.21 (0.16)^a^ | 0.17 (0.07)^a,b^ | 0.11 (0.05)^b^ | 0.01 |
| C18:1t n-9 | 0.24 (0.14)^a^ | 0.20 (0.07)^a^ | 0.13 (0.04)^b^ | 0.004 |
| C18:1t n-8 | 0.43 (0.30) | 0.38 (0.21) | 0.25 (0.14) | 0.06 |
| C18:1t n-7 | 0.34 (0.18) | 0.33 (0.10) | 0.27 (0.13) | 0.29 |
| C18:1 n-9 | 29.7 (2.44)^b^ | 32.5 (3.84)^a^ | 33.7 (2.12)^a^ | <0.001 |
| C18:1 n-7 | 1.73 (0.23) | 1.82 (0.23) | 1.68 (0.19) | 0.17 |
| C18:1 n-6 | 0.21 (0.11)^a^ | 0.20 (0.09)^a^ | 0.12 (0.05)^b^ | 0.004 |
| C18:2 n-6 | 15.9 (1.67) | 17.3 (3.20) | 17.8 (4.11) | 0.14 |
| C18:3 n-6 | 0.15 (0.07) | 0.13 (0.06) | 0.15 (0.06) | 0.67 |
| C18:3 n-3 | 0.37 (0.07) | 0.38 (0.06) | 0.39 (0.06) | 0.67 |
| C20:0 | 0.19 (0.11) | 0.23 (0.13) | 0.22 (0.07) | 0.48 |
| C20:1 n-9 | 1.23 (0.29) | 1.46 (0.40) | 1.51 (0.40) | 0.04 |
| C20:2 n-6 | 0.39 (0.08)^a^ | 0.38 (0.08)^a,b^ | 0.32 (0.06)^b^ | 0.006 |
| C20:3 n-6 | 0.53 (0.14)^a^ | 0.50 (0.16)^a,b^ | 0.41 (0.08)^b^ | 0.01 |
| C20:3 n-3 | 0.06 (0.01)^a^ | 0.06 (0.02)^a^ | 0.04 (0.01)^b^ | 0.004 |
| C20:4 n-6 | 0.49 (0.13) | 0.55 (0.14) | 0.48 (0.13) | 0.25 |
| C20:5 n-3 | 0.04 (0.02) | 0.06 (0.05) | 0.06 (0.07) | 0.47 |
| C22:0 | 0.07 (0.04) | 0.06 (0.02) | 0.08 (0.02) | 0.11 |
| C22:1 n-9 | 0.08 (0.02) | 0.07 (0.02) | 0.08 (0.02) | 0.87 |
| C22:2 n-6 | 0.06 (0.03)^a^ | 0.05 (0.01)^a,b^ | 0.04 (0.01)^b^ | 0.01 |
| C22:4 n-6 | 0.09 (0.05) | 0.08 (0.06) | 0.07 (0.05) | 0.35 |
| C22:5 n-6 | 0.05 (0.01) | 0.06 (0.03) | 0.06 (0.03) | 0.46 |
| C22:5 n-3 | 0.14 (0.03) | 0.17 (0.06) | 0.13 (0.06) | 0.05 |
| C22:6 n-3 | 0.22 (0.10) | 0.31 (0.26) | 0.24 (0.20) | 0.36 |
| C24:0 | 0.06 (0.03) | 0.04 (0.01) | 0.05 (0.01) | 0.06 |
| C24:1 n-9 | 0.08 (0.06) | 0.09 (0.07) | 0.06 (0.04) | 0.51 |
| Total Milk Fat (g/dL) | 5.5 (1.8) | 4.8 (1.7) | 3.6 (1.9) | 0.01 |

^1^Milk production groups were defined as very low (VL): <300 mL/d, moderate (MOD): > 300mL/d, and Control: exclusively breastfeeding infants with healthy weight gain.

^3^Group differences for were analyzed by one-factor ANOVA with *post hoc* Bonferroni pairwise comparisons. Group median values with different letters were statistically different (p<0.05).

**Supplemental Figure 2. Fatty acid differences by group.** Milk production groups were defined as Very Low: <300 mL/d, Moderate: > 300mL/d, and Control: externally recruited controls who were exclusively breastfeeding infants with healthy weight gain. Individual values and group means are graphed in both serum and milk for A) saturated fatty acids (SFA), B) monounsaturated fatty acids (MUFA), C) polyunsaturated fatty acids (PUFA), D) omega-6 (n-6) PUFA, E) omega-3 (n-3) PUFA, F) oleic acid (C18:1 n-9). Group differences were assessed using one-factor ANOVA with *post hoc* Bonferroni pairwise comparisons. Group means with different letters were statistically different (p<0.05).
